# Insights Into the Role of Bmi‐1 Deregulation in Promoting Stemness and Therapy Resistance in Glioblastoma: A Narrative Review

**DOI:** 10.1002/cam4.70566

**Published:** 2025-01-10

**Authors:** Fatima Shaalan, Nissrine Ballout, Wafaa Takash Chamoun

**Affiliations:** ^1^ Faculty of Medical Sciences, Neuroscience Research Center Lebanese University Hadath Lebanon

**Keywords:** Bmi‐1, epigenetics, glioblastoma, PRC1, signaling pathways, stemness, therapy resistance

## Abstract

**Background:**

Glioblastoma (GBM) is the most common primary brain tumor in adults and has a median survival of less than 15 months. Advancements in the field of epigenetics have expanded our understanding of cancer biology and helped explain the molecular heterogeneity of these tumors. B‐cell‐specific Moloney murine leukemia virus insertion site‐1 (Bmi‐1) is a member of the highly conserved polycomb group (PcG) protein family that acts as a transcriptional repressor of multiple genes, including those that determine cell proliferation and differentiation. We hereby aim to explore the specific involvement of Bmi‐1 in glioma pathogenesis.

**Methods:**

A comprehensive narrative review was employed using “PubMed”. Articles were screened for relevance specific keywords and medical subject headings (MeSH) terms related to the topic combined with Boolean operators (AND, OR). Keywords and MeSH terms included the following: “glioma”, “polycomb repressive complex 1”, and “Bmi1”.

**Results:**

In GBMs, several reports have shown that Bmi‐1 is overexpressed and might serve as a prognostic biomarker. We find that Bmi‐1 participates in regulating the gene expression and chromatin structure of several tumor suppressor genes or cell cycle inhibitors. Bmi‐1 has a critical role in modulating the tumor microenvironment to support the plasticity of GBM stem cells.We explore Bmi‐1's involvement in maintaining glioma stem cell (GSC) proliferation and senescence evasion upon regulating the chromatin structure of several tumor suppressor genes, cell cycle inhibitors, or stem cell genes in tumor cells. Additionally, we analyze Bmi‐1's involvement in modulating the DNA repair machinery or activating anti‐apoptotic pathways to confer therapy resistance. Importantly, our research discusses the importance of targeting Bmi‐1 that could be a promising therapeutic target for GBM treatment. Bmi‐1 activates and interacts with NF‐κB to promote angiogenesis and invasion, regulates the INK4a‐ARF locus, and interacts with various microRNAs to influence tumor progression and proliferation. In addition, Bmi‐1 confers radioresistance and chemotherapy by promoting cell senescence evasion and DNA repair.

**Conclusion:**

Bmi‐1 regulates self‐renewal, proliferation, and differentiation of GBM cells, promoting stemness and therapy resistance. Targeting Bmi‐1 could be a promising novel therapeutic strategy for GBM treatment.

**Abbreviations**
Bmi‐1: B cell‐specific Moloney murine leukemia virus insertion site‐1
CIC: cancer‐initiating cell
CSC: cancer stem cell
Ezh2: enhancer of zeste homolog 2
GBM: glioblastoma multiforme
GIC: glioma initiating cell
GSC: glioma stem cell
LncRNA: long noncoding RNAs
miR: microRNAs
NF‐κB: nuclear factor kappa B
NSC: neural stem cell
PcG: polycomb group
PRC: polycomb repressive complex

## 1 Introduction

Glioblastoma multiforme (GBM), the most common and aggressive primary brain cancer in adults, has a median survival rate of 11–20 months [1]. Recent data indicate that GBM tumor cells originate from neural stem cells (NSCs) in the subventricular zone (SVZ) and are associated with progression and recurrence [2]. GBM represents approximately 48% of all primary malignant central nervous system (CNS) tumors and 58% of all gliomas, making it the most common form of glioma [3].

According to the fifth edition of the World Health Organization (WHO) classification of CNS tumors, adult‐type diffuse gliomas are categorized into oligodendroglioma isocitrate dehydrogenase (IDH)‐mutant and 1p/19q co‐deleted, astrocytoma IDH‐mutant, and glioblastoma IDH‐wild type, with the latter having the worst prognosis [4]. The molecular profiling of brain tumors correlates clinical features with the disruption of specific signaling pathways during the development and progression of glial tumors, including the epidermal growth factor receptor (*EGFR*) gene, p53 (85% of GBM patients), retinoblastoma (Rb) (78% of GBM patients), and phosphatase and tensin homolog (*PTEN*) (30%–40% of GBM) [5, 6, 7]. In addition to mutations in the signature oncogenes of GBM, chromatin‐modifier genes harbor over 40% of tumors [6]. This adds much complexity to GBM's molecular profiling and suggests a role for chromatin organization in GBM pathology, which has been described for several cancer types.

## 2 Correlation Between Epigenetics and GBM

Epigenetic discoveries and their interaction with the genome have expanded our understanding of cancer biology. They identified significant genes involved in tumorigenesis, cancer progression, and chemotherapy resistance [8]. Such genome changes are important during normal mammalian development and embryonic stem cell differentiation, through which they participate in chromatin remodeling and confer exquisite plasticity to the genetic apparatus [9, 10]. The most predominant epigenetic alterations are DNA methylation and post‐translational modifications (PTMs) of histones, mostly histone methylation of H3 and H4 [9]. Analysis of epigenetic changes from The Cancer Genome Atlas (TCGA) samples identified the existence of concerted hypermethylated loci in subtypes GBM tumors, indicating the existence of a glioma‐CpG Island Methylator Phenotype (G‐CIMP) [11]. While using DNA methylation signatures as part of the WHO brain tumor classification will improve diagnostic accuracy up to 12% of prospective cases [12], methylation alone might be insufficient to induce gene repression in certain instances. Instead, relevant chromatin remodeling forced by histone modification mechanisms may be required to predispose tumor suppressor genes to DNA hypermethylation. Such histone modifications, especially, the key PcG mark, H3K27me3, may leave the cells vulnerable to tumor cell progression [13].

**FIGURE 1:**
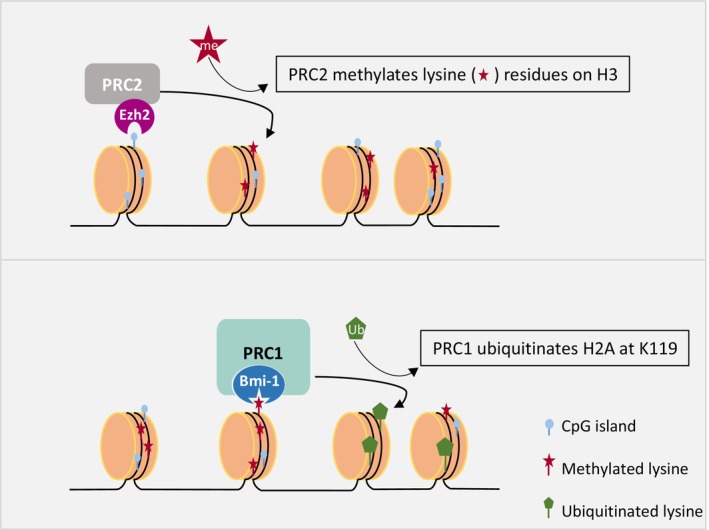
Role of polycomb repressive complexes (PRC2 and PRC1) in chromatin modification. (a) *PRC2 complex activity*. PRC2, with its catalytic subunit Ezh2, methylates lysine‐27 residues on histone H3. This methylation marks chromatin for transcriptional repression. (b) *PRC1 complex activity*. Following PRC2‐mediated methylation, the PRC1 complex, with Bmi‐1 as a core component, catalyzes histone 2A monoubiquitination and remodels the chromatin structure. This further stabilizes chromatin compaction, contributing to gene silencing.

Polycomb group (PcG) proteins, which are considered with the utmost concern in the present review, were first discovered in Drosophila. They are epigenetic gene silencers of homoeotic genes and are crucial in regulating various physiological and pathological processes, including genetic imprinting, cell cycle, cell identity, embryonic development, and oncogenesis [14, 15, 16]. PcG proteins form multi‐subunit complexes, which are divided into two main protein complexes: the polycomb repressive complex (PRC1) involved in the maintenance of gene silencing and the PRC2 involved in silencing initiation [17, 18]. Initially, PRC2 uniquely methylates lysine (K) residues on H3 (H3K27) through the catalytic subunit Ezh1/2 in a complex with other core subunits, as previewed in Figure 1a [19]. PRC1 then identifies H3K27me3, which marks repressed chromatin regions. Upon recognition, PRC1 ubiquitinates H2A at K119, leading to chromatin compaction and suppression of gene expression, as illustrated in Figure 1b. Thus, the reciprocal interaction between PRC1 and PRC2 can affect gene expression. They are key determinants of the fate of neural progenitor cells, influencing whether they self‐renew or differentiate into neurons or glial cells [20]. These functions are closely correlated with cancer development in human malignancies.

B cell‐specific Moloney murine leukemia virus integration site‐1 (Bmi‐1), a core subunit of PRC1, is essential for the proliferation of postnatal stem cells, primarily in the CNS [21]. It is required for the self‐renewal and differentiation of several adult stem cells, including NSCs of the SVZ [21, 22]. During brain development, Bmi‐1 is overexpressed in NSCs, contributing to the enhanced proliferation of undifferentiated progenitor cells during corticogenesis [23]. Several studies have identified Bmi‐1 protein overexpression in various human cancers, including prostate cancer [24], lymphoma [25], multiple myeloma [26], and primary neuroblastoma [27], and it has been considered a marker of poor prognosis. In particular, Bmi‐1 is critical for the development of the cerebellum, and its increased protein expression is frequently observed in medulloblastoma [28]. In addition, Bmi‐1 binds to coding and noncoding genes that are critical for regulating neural lineage commitment, which may be relevant to brain cancers [29].

In our present narrative review, we explored the correlation between epigenetic alterations, particularly PRC1 deregulation, and GBM pathogenesis. By synthesizing the current literature, we aimed to discuss the unique role of Bmi‐1 in regulating gene expression and chromatin structure in GBM, thereby addressing a significant gap in the literature regarding the specific involvement of PRC1, particularly Bmi‐1, in glioma pathogenesis. By providing a detailed analysis of the molecular mechanisms underlying the deregulation of Bmi‐1 and its clinical implications, this review analyzes Bmi‐1's influence in critical molecular pathways, ultimately promoting tumor growth, stemness, and resistance to treatment. We aim to provide a deeper understanding of the complex interplay between epigenetics and GBM, ultimately paving the way for developing novel targeted therapies and personalized treatment approaches for this aggressive form of brain tumor.

## 3 Methodology

For this review, an identified search strategy is employed to collect relevant studies focusing on glioblastoma and epigenetics. PubMed, a free database primarily including the MEDLINE database of references on life sciences and biomedical topics, had been searched. We used specific keywords and medical subject headings (MeSH) terms related to the topic combined with Boolean operators (AND, OR). Keywords and MeSH terms included the following: “glioma”, “polycomb repressive complex 1”, and “Bmi1”.

Sources obtained are analyzed according to specific and detailed criteria. First and most importantly, the sources should align with the purpose of the review. Further inclusion criteria for study subjects include patients diagnosed with malignant gliomas, including different grades based on the WHO classification, as well as cases involving GBM. In addition, animal models highlighting the role of PRC1 in glioma are being considered. The included studies were also to be published in peer‐reviewed journals. Conversely, the exclusion criteria excluded articles and studies focusing on nonglioma brain tumors, such as medulloblastoma. Furthermore, all studies that presented incomplete or insufficient molecular profiling information on the topic were excluded. Finally, non‐English articles were excluded.

The methodology employed in this review is a comprehensive narrative review that synthesizes the existing knowledge on gliomas, with a particular emphasis on the role of PRC1 in epigenetic regulation and potential therapeutic strategies. This method enabled us to compile and analyze data from numerous sources, including original research articles, reviews, and molecular studies, thereby providing a nuanced and holistic understanding of the subject matter. A literature search identified 174 articles. Subsequent screening based on precise inclusion and exclusion criteria resulted in the selection of 60 articles that met the predefined criteria and were thus included in the references list of our present review.

## 4 Results

**FIGURE 2:**
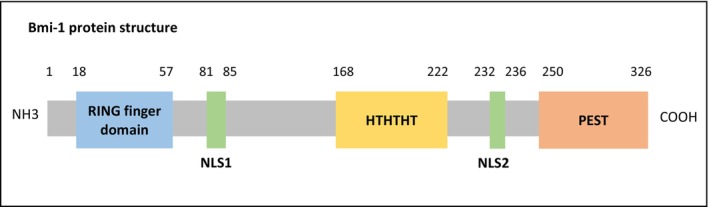
Structural domains of the Bmi‐1 protein. Bmi‐1 protein, containing 326 amino acids, comprises three main regions: The zinc finger motif (C3HC4) located in the finger domain responsible for DNA binding and transcriptional regulation, the central helix‐turn‐helix‐turn‐ helix‐turn (HTHTHT) domain facilitating E3 ubiquitin ligase activation and gene repression, and the carboxyl terminus enriched in proline (P), glutamic acid (E), serine (S), and threonine (T), which is thus known as the PEST sequence that acts as a proteolytic signal leading to protein degradation. Two nuclear localization signals NLS1 and NLS2, located between these regions, are involved in nuclear import of Bmi‐1.

Having established a comprehensive understanding of the molecular landscape of GBM and the critical role of epigenetics in this context, we present the key findings of our investigation, elucidating the intricate mechanisms by which Bmi‐1 influences critical molecular pathways, ultimately promoting tumor growth and resistance to treatment. Zhou et al. described this transcriptional repressive complex as a gene silencer during embryonic development. The Bmi‐1 protein comprises three main regions: the amino terminus, central region, and carboxyl terminus, each of which serves distinct functions. The amino terminus features a ring finger domain containing a cysteine‐rich zinc finger motif. In contrast, the carboxyl terminus is characterized by a peptide sequence rich in proline (P), glutamic acid (E), serine (S), and threonine (T), which is thus known as the PEST sequence, and the central region consists of a conserved helix‐turn‐helix‐turn (HTHTHT) domain. Two nuclear localization signal sequences (NLS1 and NLS2) are situated between these regions. A loop finger domain is also primarily involved in DNA binding and transcriptional regulation and is essential for activating E3 ubiquitin ligase activity, as shown in Figure 2 [30].

### 4.1 Expression

Bmi‐1 has emerged as an important molecule in medical research because it can initiate cells that promote the undifferentiated state of GBM cells. The incidence of Bmi‐1 positivity is augmented in grades II‐IV in human gliomas, and Bmi‐1‐positive cells frequently hold clinical importance in prognostic evaluations of overall survival in patients with glioma [31, 32, 33]. Among the three molecular subclasses of GBM, classical, proneural, and mesenchymal, studies have indicated an augmented expression of Bmi‐1 in mesenchymal GBM cells compared with proneural cells, suggesting a role in molecular subtype transitions [34]. Although Bmi‐1 expression alone may not serve as a significant prognostic indicator in astrocytic tumors, Tirabosco et al. identified the disruption of the Bmi‐1/p16 pathway as a common molecular mechanism with plausible prognostic implications [35]. Correspondingly, markedly elevated mRNA and protein levels of Bmi‐1 suggest a potential role for Bmi‐1 in the aggressive nature of GBM [36]. Although some studies have shown that Bmi‐1 is expressed more in high‐grade than low‐grade gliomas [37], others have noted varying levels of Bmi‐1 expression across glioma grades without a direct correlation with patient's survival. These opposing findings highlight the complexities of Bmi‐1's prognostic role in gliomas [38]. Furthermore, it is worth noting that Bmi‐1 overexpression in stem/progenitor cells is more pronounced in culture and insufficient to cause CNS stem/progenitor cells to become tumorigenic in vivo without other pronounced mutations [39].

### 4.2 Transcriptional Regulation

The roles of various transcription factors have been described in the development and maintenance of brain tumors, particularly in GBM. For instance, SOX7, as first described by Takash et al., modulates the activity of the Wnt/β‐catenin signaling pathway, thereby influencing the proliferation and invasion of glioma cells [40, 41]. Concerning Bmi‐1, several candidate genes are proclaimed to be upstream and might be responsible for Bmi‐1's overexpression, as summarized in Table 1. For example, Aldaz et al. showed that the SOX9 transcription factor positively modulates Bmi‐1 expression, enhancing tumor cell survival and proliferation in GBM by directly binding to the promoter of Bmi‐1. This regulatory axis influences cellular senescence and apoptosis, potentially via the repression of tumor suppressor genes like p21CIP [42]. Similarly, the oncogene c‐Myc directly regulates Bmi‐1 expression through c‐Myc binding to an E‐box in its promoter. This suggests that Bmi‐1 overexpression in patients with GBM might be a result of c‐Myc activation, which in turn cooperates with the c‐Myc oncogene to promote tumorigenesis and maintain key properties of GBM cells [43]. Nevertheless, Bmi‐1 has been proven to be a functional target of nuclear‐encoded cytochrome c oxidase subunit 4 isoform 1 (COX4‐1) in NSC and potentially contributes to GBM pathogenesis [44]. Because Bmi‐1‐overexpressing cells preferentially localize to hypoxic regions in brain tumor areas, Qiu et al. found that the expression of Bmi‐1 is controlled by hypoxia‐inducible‐factor 1 α (HIF‐1α), and binding sites exist between HIF‐1α and Bmi‐1 [45, 46]. Independent studies have demonstrated that sonic hedgehog (Shh) signaling regulates Bmi‐1 expression in gliomas. Gli1 and Gli2 control the expression of Bmi‐1 by their binding to the Bmi‐1 promoter and eventually upregulating multidrug‐resistant proteins that promote chemoresistance in glioma cells [47, 48].

**TABLE 1:** Factors regulating Bmi‐1 at the transcriptional level.

Factor	Function	References
c‐Myc	Increases Bmi‐1 expression by binding to the E‐box sequence in the Bmi‐1 promoter	[43]
SOX9	Modulates Bmi‐1 expression by binding to its promoter	[42]
HIF‐1 α	Directly regulates the upregulation of the Bmi‐1 expression level	[45]
COX4‐1	Increases Bmi‐1 expression at the mRNA and protein levels	[44]
GLI1	Directly binds to the promoter region of Bmi‐1 in glioma cells	[47]
GLI2	Acts upstream of Bmi‐1 and influences its expression by binding to the promoter	[48]
HDAC	Induces Bmi‐1 promoter activity by regulating Sp1	[38]

Abbreviations: COX4‐1, cytochrome c oxidase subunit 4 isoform 1; Gli, glioma‐associated oncogene homolog; HDAC, histone deacetylase; HIF‐1 α, hypoxia‐inducible‐factor 1 α; SOX9, SRY‐Box Transcription Factor 9.

Some studies reported post‐translational modifications of Bmi‐1 that promoted protein stabilization and enhanced activity. For instance, interleukin‐17A (IL‐17A), produced by the main cytokine effector of T helper cells promotes Bmi‐1 stabilization in GBM cells as a result of the activation of the PI3K/AKT pathway [49]. Moreover, ubiquitin‐specific protease 22 (USP22) interacts with Bmi‐1 to ensure its deubiquitination for post‐translational stabilization. Both USP22 and Bmi‐1 regulate a series of genes involved in glioma stemness [46, 50].

### 4.3 Tumorigenesis

Bmi‐1 is an oncoprotein prerequisite for transformation, metastatic migration, and malignancy maintenance in multiple cancer models. As tumorigenesis is a multi‐gene and multi‐mechanism process, around Bmi‐1, several gene pathways are involved in its regulatory mechanism, including MMP‐2, MMP‐9, NF‐kB, VEGF‐C, and the INK4a/ARF pathway, as mentioned in Table 2.

**TABLE 2:** Downstream effectors of Bmi‐1.

Factor	Function	References
NF‐κB	Activation of NF‐κB leads to subsequent upregulation and activation of MMP‐2/MMP‐3/MMP‐9 and VEGF‐C	([51]; [52]; [53]; [54])
P21	Bmi‐1 directly suppresses its expression	[42]
P16	Bmi‐1 regulates glioma cell invasion through modulation of p16 expression	[55]
EfnA5	Bmi‐1 mediates the regulation of EfnA5 via modulation of H3K27me3 levels at its promoter	[34]

Abbreviations: EfnA5, ephrin A; NF‐κB, nuclear factor kappa B.

#### 4.3.1 Bmi‐1 in Maintaining the Stemness State in GBM

Cells with “stem‐like” properties have been described in many cancers. They exhibit a stem cell‐like chromatin structure essential for tumor proliferation and frequently express stem cell genes. Cancer stem cells (CSCs) possess extensive self‐renewal and differentiation capabilities and are commonly present in most tumors. They contribute to the diversity of cancer cells and are characterized by their capacity for self‐renewal, multipotency, and the ability to initiate tumor formation [56, 57]. Glioma stem cells (GSCs), identified using the cell surface marker CD133, can develop during transformation, and differentiated non‐stem cancer cells can undifferentiate into CSCs. Ample evidence shows that CSCs or cancer‐initiating cells (CICs) play important roles in GBM [58, 59]. GSCs actively remodel their environment to support the plasticity of GBM stem cells by promoting angiogenesis and other tumorigenic characteristics [60].

It is widely believed that the INK4a/ARF tumor suppressor locus is a critical downstream target of Bmi‐1. Interestingly, the promotion of Bmi‐1 migration and invasion by glioma cells is probably mediated by the regulation of p16, a protein that can suppress the proliferation and self‐renewal of glioma cells [55]. In fact, Bmi‐1 is upregulated and p16 is downregulated in 73.5% of cases [35]. In contrast, Bruggeman et al. found that INK4A/ARF is frequently deleted in GBM tumors so that Bmi‐1 oncogenic function might be independent of a functional INK4A/ARF locus. For instance, in the absence of functional INK4a/ARF, Bmi‐1‐deficient cells exhibit reduced differentiation, rapid tumor growth, and a shift toward a less malignant phenotype [61].

Beyond the repressive effect of Bmi‐1 on the INK4a/ARF tumor suppressor locus, its role might be involved in regulating differentiation pathways to maintain the stem cell niche and influence tumor (stem) cell behavior [62]. As Bmi‐1 is found to be highly enriched in CD133‐positive CICs [36], it plays a vital role in maintaining the clonogenic potential of CD133‐positive cancer stem‐like cells in GBM by promoting self‐renewal and proliferation [62]. Indeed, Bmi‐1 regulates the expression of genes involved in stem cell maintenance, differentiation, and tumorigenesis. Further investigations by Abdouh et al. showed that Bmi‐1 plays a crucial role in maintaining GSC proliferation and survival by interacting with multiple tumor suppressor pathways to preserve their undifferentiated state. Bmi‐1 also tends to prevent apoptosis in CD133‐positive cells, thereby ensuring their survival and continued renewal. This anti‐apoptotic effect promotes the survival of stem cells within the tumor environment primarily through Bmi‐1 repression of p21Cip, a target of p53 [62]. Subsequently, Bmi‐1 inhibits the expression of tumor suppressors, contributes to tumor malignancy, and facilitates the evasion of senescence in GSCs [42]. In addition, previous studies have found that conditions such as hypoxia, nutrient restriction, and acidic stress promote the maintenance of GSCs. Through regulatory mechanisms, Bmi‐1 may remodel the tumor microenvironment and make it a well‐suitable environment for GSCs to maintain the undifferentiated state of glioma initiating cells (GICs). Uniquely, differential activation of Bmi‐1 in CD‐133‐positive cells governs key downstream effectors, mainly metabolism‐associated processes (gluconeogenesis and TCA cycle). These results were proven through gene clustering analysis, which revealed distinct tumor‐propagating roles for Bmi‐1, depending on the cell's CD133 status [63]. Surprisingly, in cultured GBM tumor cells, Bmi‐1 mRNA expression was significantly higher in CD133‐negative than in CD133‐positive cells. Thus, Bmi‐1 would not only promote its known regulatory role in CD133‐positive stem cells but also possess an uncharacterized role in CD133‐negative late progenitor populations [64].

More research to the field of GSCs reveals an increasing number of molecules/pathways [e.g., bone morphogenetic proteins and transforming growth factor β (TGF‐β/BMP) or the endoplasmic reticulum (ER) stress pathway] involved in the maintenance and differentiation, ultimately causing the repression of tumor suppressor genes. Bmi‐1 modulates gene expression patterns associated with self‐renewal and differentiation dependent on the cellular response to the TGF‐β/BMP and ER stress pathways. Such signals contribute to the aggressive behavior of GBM and the persisting CSC characteristics [29]. On the other hand, the role of reactive oxygen species (ROS) in CSCs has been characterized in the control of stem‐like GICs and their tumor‐initiating capacity. In fact, induced oxidative stress triggers the activation of p38 MAPK in GICs. Once activated, p38 MAPK initiates downstream signaling cascades that result in the degradation of Bmi‐1 protein, by its phosphorylation in a MAPK‐dependent manner [65]. Subsequently, the initial downregulation of Bmi‐1 may trigger the upregulation of differentiation markers in GICs. In the same context, silencing Bmi‐1 in pediatric GBM models suppresses cell proliferation and neurosphere formation in vitro, abrogates tumor formation in mice brains in vivo, and eliminates the tumor‐forming capacity of pediatric GBM stem cells, emphasizing the complexity of epigenetic regulation in this context [66]. Taken together, these data suggest that Bmi‐1 plays a crucial role in preserving the stem cell properties of GICs.

The Eph/ephrin family plays a significant role in cell adhesion, cell behavior, migration, and differentiation during embryonic development and homeostasis. The ephrin proteins are frequently deregulated in GBM, where they suppress regenerative processes and promote angiogenesis, invasion, and migration to GSCs [67]. Epigenetic regulators appear to influence Ephrin expression in GBM, mainly through PcG proteins. By enhancing H3K27me3 at the EfnA5 promoter locus, Bmi‐1 regulates and reinforces EfnA5 repression to promote proliferation, invasion, and tumor formation in GIC in both mouse and human models [34]. This suggests a tightly related polycomb feedforward loop for gliogenesis. In addition, the DHHC (Asp‐His‐His‐Cys)‐S‐acyltransferase protein family targets different GSC subsets in specific niches and regulates the plasticity of cells in these subtypes via its interaction with Bmi‐1 to regulate its polyubiquitination, thereby influencing the plasticity of GBM stem cells [68].

Screening of several key proteins controlling cell cycle, development, metabolism, apoptosis, and growth showed that downregulation of Bmi‐1 reduced p‐AKT, Nestin, Bcl2, and GSK3b protein levels and induced differentiation in cancer cells, as well as decreased proliferation, survival, migration, and clonogenicity [69]. In a study of the Bmi‐1 interactome in GBM, Bmi‐1 interacts with PRC1 subunits as RING1A/B proteins via its ring domain to promote its E3 ubiquitin ligase activity on H2A and enhance Bmi‐1's stabilization. This interaction is believed to result in transcriptional repression and carcinogenic activity primarily. Furthermore, studies have demonstrated that Bmi‐1 regulates mRNA splicing events in GBM cells. Bmi‐1 has been identified as an alternative splicing regulator that regulates the expression of genes associated with the splicing machinery as well as alternative splicing events. Bmi‐1 splicing dysregulation may result in the creation of carcinogenic protein isoforms or changes in gene expression patterns, both of which could contribute to cancer formation. For example, Bmi‐1 affects the expression of genes involved in cholesterol production and transport as well as alternative splicing events related to cholesterol metabolism, thereby modulating cellular lipid homeostasis and metabolic processes critical for tumor growth and survival [70].

It is worth noting that although Bmi‐1 induces significant proliferation and self‐renewal by remodeling the tumor cells surrounding the microenvironment, Bmi‐1 is incapable alone of promoting tumor formation without another mutation. One possible reason for the need for a favorable chromatin configuration is the complete recapitulation of the epigenetic landscape necessary for tumorigenesis [63].

#### 4.3.2 Bmi‐1 and microRNAs

In recent years, microRNAs (miRNAs), a group of small and noncoding RNAs, have emerged as potential and promising markers for cancer diagnosis and targeted therapy. Several studies have proved a link between miR‐128 and the self‐renewal factor Bmi‐1 and loss of GSC self‐renewal. MiR‐128's direct regulation of Bmi‐1 by miR‐128 in SVZ‐derived NSCs decreases glioma cell proliferation in vitro and in vivo and reduces GSC self‐renewal capacity [71]. However, in GBM, epigenetic methylation of one of the three CpG islands in miR‐128 contributes to its downregulation of normally high miR‐128 expression in GSCs, which is correlated with the upregulation of Bmi‐1 [72, 73]. Clinically, miR‐128 expression correlates with improved overall survival in patients with GBM, and upregulation of miR‐128 decreases H3K27me3 histone methylation and increases p21/Cip1 levels, consistent with Bmi‐1 downregulation [71, 74].

In glioma cells, miR‐218 expression is drastically downregulated. Mechanistic investigations identified Bmi‐1 as a functional downstream target of miR‐218, through which miR‐218 ablates cell migration, proliferation, and self‐renewal by regulating genes involved in tumor development (HIF‐1a and PI3K/Akt signaling pathways); it also regulates tumor metabolism, particularly hexokinase‐2 [75, 76]. On the other hand, miR‐194, miR‐429, and miR‐340 demonstrate tumor suppressive properties in GBM by targeting Bmi‐1 to suppress glioma cell migration, invasion, and epithelial‐to‐mesenchymal transition [77, 78, 79]. Interestingly, long noncoding RNAs (lncRNAs) such as DANCR and LINC00152 sponged miR‐135a‐5p and miR‐16, respectively, to reverse the inhibitory effects on glioma progression by promoting Bmi‐1 expression and Bmi‐1‐mediated effects on glioma cell proliferation and invasion [80, 81].

Taken together, the data presented here offer an interpretative framework for the mechanism by which PRC1 through Bmi‐1 modulates key cellular mechanisms either directly or indirectly and its ability to modulate the tumor microenvironment, rendering it suitable for GSCs, promoting tumor formation, proliferation, and senescence evasion. These results are summarized in Figure 1.

### 4.4 Bmi‐1 Confers Resistance to Therapy in Glioma Cells

In addition to its role in driving the pathogenesis of GBM, accumulating evidence suggests that Bmi‐1 plays a crucial role in promoting resistance to conventional therapeutic interventions. Resistance to therapy presents a critical challenge in the management of GBM, contributing to treatment failure and disease recurrence.

The classic standard‐of‐care protocol for treating GBM involves surgical resection of the tumor to the maximal feasible extent, followed by radiotherapy and temozolomide chemotherapy [82]. However, malignant glioma cells can resist cytotoxic treatment‐induced cell death upon the upregulation of the oncoprotein Bmi‐1 during glioma progression [53]. The microenvironment surrounding glioma cells plays a critical role in determining their response to chemotherapy. Endothelial cells, in particular, can promote the aggregation of GSCs into neurospheres and influence the organization of the microenvironment surrounding GSCs, particularly because stem cell biomarkers are enriched in these cells as Bmi‐1 [83]. Another mechanism contributing to Bmi‐1‐induced therapy resistance is the direct binding of Gli1 to the promoter region of Bmi‐1. This would further upregulate the expression of multidrug resistance‐associated protein‐1, thus promoting chemoresistance in glioma cells [47]. Therefore, the science community must study the molecular pathways that contribute to chemoresistance.

**FIGURE 3:**
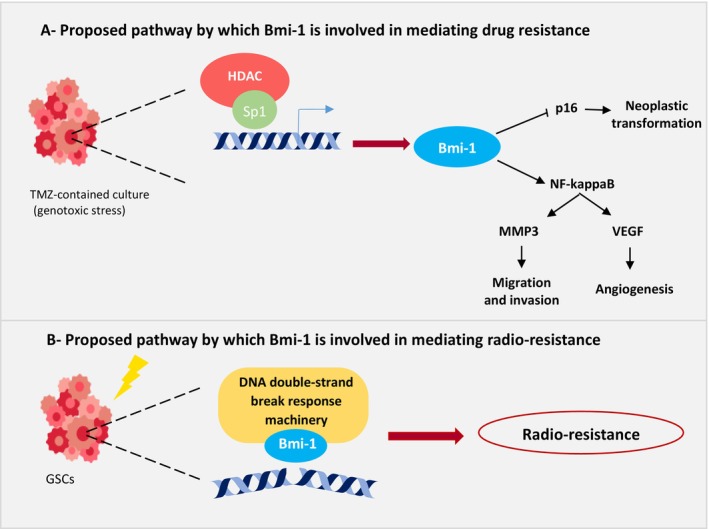
Proposed mechanisms by which Bmi‐1 mediates drug and radio‐resistance in GSCs. (A) Bmi‐1 promotes drug resistance under stress conditions. Both tumor microenvironment‐induced stress and chemotherapy‐induced genotoxicity from temozolomide (TMZ) upregulate the transcription factors HDAC and Sp1, leading to Bmi‐1 activation. Bmi‐1 activation subsequently triggers several downstream effects, including the repression of p16, which contributes to neoplastic transformation and the activation of NF‐kappaB. NF‐kappaB further induces MMP3 gene expression, thus enhancing migration and invasion, and VEGF, promoting angiogenesis and neovascularization. (B) *Bmi‐1 mediates radio‐resistance following ionizing radiation (IR) in GSCs*. IR activates DNA response machinery, which in turn leads to Bmi‐1 upregulation. This increase in Bmi‐1 expression promotes cellular survival and radio‐resistance in GSCs.

In glioma, GBM specifically, drug resistance is linked to dysregulation of several key signaling pathways, for instance, the nuclear factor kappa B (NF‐κB) signaling pathway [84]. Bmi‐1 tends to protect glioma cells against cytotoxic reagent‐induced cell death via its activation of the NF‐κB‐mediated anti‐apoptotic pathway. Bmi‐1, through the induction of phosphorylation of IKKb and IκBa and co‐localization with the catalytic subunit of NF‐κB in the cell nucleus of tumor cells, activates NF‐κB and renders glioma cells resistant to cytotoxic treatments [53]. This activation would then lead to subsequent activation of downstream effectors that participate in the migration and invasiveness of glioma cells, in particular, matrix metalloproteinase‐3 (MMP‐3) [54]. As a result of MMP‐3 activation via the Bmi‐1/NF‐κB axis, Bmi‐1 participates in the activation of several other MMPs, including MMP‐1, MMP‐7, MMP‐8, MMP‐9, and MMP‐13. In particular, MMP‐9 plays an essential role in the invasiveness of glioma cells because it directly degrades basal membrane and extracellular matrix proteins [52, 85]. Additionally, Bmi‐1 promotes neovascularization by upregulating VEGF‐C expression in glioma cells upon NF‐kB activation [51]. Taken together, these results suggest a mechanism by which Bmi‐1 stimulates NF‐κB‐pathway activation considering that such a pathway is constitutively activated in GBM to maintain GSC survival and proliferation (see Figure 3A).

A growing body of evidence has shown that Bmi‐1 upregulation in tumor cells is often associated with chemotherapy resistance, and its silencing can induce notable apoptosis and enhance chemotherapy effectiveness [86]. Indeed, Bmi‐1 overexpression promotes tumor growth and proliferation and is usually correlated with poor prognosis and high‐grade gliomas [32, 86]. Instead, Tsai et al. showed that the HDAC/Sp1/Bmi‐1 axis is a more accurate indicator of tumor malignancy and patient survival [38]. Essential for Bmi‐1 expression, HDAC activity can be targeted using vorinostat glioma xenografts [38]. Recent studies have highlighted the role of Phragmunis A (PGA), which is derived from the fruitbody of *Trogia venenata*, in inducing temozolomide‐resistant GBM cell apoptosis. This is a complex mechanism by which PGA inhibits specific pathways leading to reduced Bmi‐1 expression. Ultimately, this leads to GBM cell apoptosis and overcomes drug resistance by reducing anti‐apoptotic proteins known to confer chemoresistance to cancer cells [38]. Targeting Bmi‐1 expression with agents such as vorinostat and PGA shows promise in overcoming drug resistance; however, the translational potential of these findings necessitates validation in clinical trials.

Radiotherapy is a crucial therapeutic approach for treating malignant tumors. It is estimated that approximately 92% of patients with nervous system malignancies require radiotherapy at some point during their treatment, compared with 50% of patients with other malignant tumors [87]. Unfortunately, some GBMs are relatively radioresistant, resulting in poor patient outcomes. Because Bmi‐1 is enriched in CD133‐positive cells compared with CD133‐negative cells [36, 88], it is proposed that Bmi‐1 plays a role in the resistance of GSCs to radiation and helps cells evade cell cycle arrest. Upon exposure to radiation, Bmi‐1 expression is upregulated, particularly at doses of 6 Gy or higher, because of the downregulation of miRNA‐128 [89, 90]. One plausible explanation is Bmi‐1's involvement in suppressing apoptotic‐related genes in tumor cells, thus contributing to the evasion of senescence and maintenance of cell proliferation [62, 69]. In contrast, Facchino et al. revealed that Bmi‐1 might be enriched in the chromatin fraction rather than upregulated. This leads to the activation of the DNA double‐strand break response machinery, and tumor cells become radioresistant in CD133‐positive GBM cells [91] (see Figure 3B). As a result, Bmi‐1 activates both anti‐apoptotic pathways at the transcriptional (repression of tumor suppressor genes) and non‐transcriptional levels (activation of DNA repair machinery) [62, 91]. Although Bmi‐1 inhibition has the potential to decrease self‐renewal capacity and induce apoptosis in CSCs, its clinical efficacy and safety in GBM treatment remain to be fully elucidated.

## 5 Discussion

The findings of our narrative review offer insights into Bmi‐1's intricate role in deregulating epigenetic mechanisms and chromatin structure in GBM. Our analysis revealed Bmi‐1's integral role in altering signaling pathways associated with tumor development and progression. Intriguingly, these results further highlight Bmi‐1's role in GBM progression, proliferation, senescence evasion, and therapy resistance. Our comprehensive review also provides an explanation of the mechanistic basis of Bmi‐1‐mediated epigenetic changes that promote the stemness state of tumor cells and it proposes Bmi‐1 as a possible prognostic biomarker of GBM.

Chemical inhibition of Bmi‐1 reduces self‐renewal and metastatic potential and decreases the viability of mesenchymal GSCs under stress [46, 92]. In GBM, Bmi‐1 enhances self‐renewal and proliferation by repressing tumor suppressor genes and cell cycle inhibitors, such as p16, p19, and p21, to promote tumor cell undifferentiation [35, 42]. Because Bmi‐1 is essential for tumor cell growth, its downregulation is hypothesized to contribute to the aging of GBM cells and promote their senescence [93]. Interestingly, it has been suggested that if Bmi‐1 is downregulated or silenced, it would enhance the effectiveness of chemotherapy at lower concentrations while reducing the adverse effects of chemotherapy [86].

Recently, the small‐molecule Bmi‐1 inhibitor PTC209, which exhibits selective post‐transcriptional properties, has shown promising therapeutic results in suppressing Bmi‐1 expression and mitigating self‐renewal in brain tumor cells [94, 95]. PTC209 potential effects are attributed to the inhibition of a subset of Bmi‐1‐targeted tumor suppressor genes, the induction of apoptosis, the enhancement of p53 expression, and the regulation of the serine/ threonine‐protein kinases AKT as well as the JNK pathway [96, 97]. Interestingly, it holds the potential to sensitize tumor cells to radiotherapy, at least by impeding the DNA damage response [91, 95]. However, the blood–brain barrier permeability of PTC209 was not high under normal conditions. To overcome this limitation, Poonaki et al. investigated the effect of PTC209 loaded into a nanocarrier‐based drug delivery (PLGA–PEG) nanoparticle conjugated with CD133 antibody. Nano‐PTC209 considerably inhibited GSC migration/invasiveness, presumably by inducing apoptosis and cell cycle arrest [97].

Another small molecule identified as a potent repressor of Bmi‐1 is PTC596 [98]. PTC596 modulates Bmi‐1 via post‐translational phosphorylation, leading to protein degradation. Studies have revealed that the combination of PTC596 with radiotherapy is a promising novel therapy for children with diffuse intrinsic pontine glioma (DIPG) [99]. Molecular analyses revealed that PTC596 targeted not only Bmi‐1 but also Ezh2, potentially explaining the modified phenotype observed as a result of EMT in GBM cells [100]. In addition, PTC596, by modulating Bmi‐1, could efficiently prevent GBM colony growth and significantly extend lifespan [46]. It is currently being tested on recently diagnosed children with DIPG (study ID: NCT03605550) and has completed phase I clinical trials in adults with advanced solid tumors (NCT02404480).

By focusing on epigenetic alterations, particularly PRC1/Bmi‐1 deregulation, our present review does address a crucial and emerging area of cancer research, highlighting the significance of epigenetic factors in GBM pathogenesis. However, as a narrative review, there may be selection bias in the included studies, and the review may not fully address the variability in study quality or the heterogeneity of the existing research. While focusing on Bmi‐1 might help understand some mechanisms in GBM, it limits the scope, potentially overlooking other significant uncovered epigenetic mechanisms and processes that could provide a better understanding of the disease. On the other hand, a detailed examination of Bmi‐1's role in GBM pathogenesis positions it within a broader context of epigenetic regulation by PcG proteins. Our comprehensive work complements the existing research on PRC2, particularly the extensively studied EZH2 subunit, which is known for its involvement in the epigenetic silencing of tumor suppressor genes and promotion of cancer progression [101]. The comparison of Bmi‐1 and EZH2 underscores the potential of targeting these epigenetic regulators in GBM therapy [33]. This narrative review highlights the unique contribution of Bmi‐1 among PRC1 subunits, providing a nuanced perspective on its role in GBM. Furthermore, our understanding of Bmi‐1's function in preserving stemness and promoting therapy resistance aligns with emerging investigations into GSCs and their regulation by epigenetic modifiers. Therefore, we propose Bmi‐1 as a potential prognostic marker that can influence clinical approaches and improve patient's outcomes.

## 6 Conclusion

Despite all the progress in medicine, patients with GBM still have the lowest median survival, likely because this tumor rapidly becomes radio‐chemo‐resistant and infiltrates the surrounding brain tissue. Epigenetic modifications are gaining strong relevance in glioblastoma because they can be clinical biomarkers and potential drug targets. In GBM, Bmi‐1's aberrant expression level explains the activation of self‐renewal pathways upon interacting with multiple tumor suppressor pathways to preserve GSCs undifferentiated state. Bmi‐1 would also modulate gene expression patterns associated with self‐renewal and differentiation dependent on the cellular response to stress conditions that correlates its upregulation to the aggressive behavior of GBM as well as to the resistant nature of GSCs. Our thorough analysis highlighted several critical mechanisms through which Bmi‐1 exerts its oncogenic function, enhances the stemness state, and confers resistance to conventional therapies.

Regardless of advancements in understanding Bmi‐1's contributions to cancer, there remains a significant gap in the literature regarding its role in promoting stemness and how it contributes to therapy resistance. Although research on Bmi‐1's contribution in GBM cells explained some of the mechanisms that contributed to its aggressive nature, targeting Bmi‐1 alone could not serve as an optimal option against GBM. Since Bmi‐1's major role is in remodeling the tumor niche to support the plasticity of GSCs rather than initiating the tumorous mass, we propose that silencing Bmi‐1 should be along with other treatment(s) to obtain the maximum benefit. Targeting epigenetic mechanisms in GBM holds great promise and has provided optimistic results in many preclinical studies. Further research is certainly required to comprehend the precise mechanism(s) dictating the epigenome to participate in promoting tumor growth, senescence evasion, and therapy resistance to help discover novel therapeutic options for brain tumors.

## Author Contributions

F.S. conceived the study, performed the literature search, curated the data, and wrote the original draft. N.B. contributed to proofreading and validation. W.T.C. developed the methodology, carried out the analysis, reviewed and edited the manuscript, and supervised the work.

## Ethics Statement

The authors have nothing to report.

## Conflicts of Interest

We hereby confirm that there are no known conflicts of interest associated with this publication and the authors did not receive support from any organization for the submitted work. We confirm that the manuscript has been read and approved by all named authors and that there are no other persons who satisfied the criteria for authorship but are not listed. This article does not contain any studies with human or animal subjects performed by any of the authors.

## Data Availability

All data mentioned in this study had been extracted from cited articles.
